# Editorial: Digital strategies to reduce salt consumption

**DOI:** 10.3389/fpubh.2023.1244216

**Published:** 2023-10-04

**Authors:** Sonu M. M. Bhaskar

**Affiliations:** ^1^Global Health Neurology Lab, Sydney, NSW, Australia; ^2^Neurovascular Imaging Laboratory, Clinical Sciences Stream, Ingham Institute for Applied Medical Research, Liverpool, NSW, Australia; ^3^Department of Neurology and Neurophysiology, Liverpool Hospital and South West Sydney Local Health District, Liverpool, NSW, Australia; ^4^NSW Brain Clot Bank, NSW Health Pathology, Sydney, NSW, Australia; ^5^Stroke and Neurology Research Group, Ingham Institute for Applied Medical Research, Liverpool, NSW, Australia; ^6^Department of Neurology, National Cerebral and Cardiovascular Center, Suita, Japan

**Keywords:** digital health, digital public health, salt consumption, global health, cardiovascular diseases, stroke

Salt consumption has become a pressing public health concern worldwide, with most individuals consuming double the daily recommended amount set by the World Health Organization (WHO) (1). This increase can be attributed to the proliferation of ultra-processed foods and lifestyle changes that prioritize convenience over health (2). The consequences of high salt consumption are dire, leading to preventable non-communicable diseases such as high blood pressure, cardiovascular disease, and stroke (3).

Addressing the challenge of reducing salt consumption requires concerted efforts at both the individual and population levels (4). While individuals must take responsibility for their dietary choices, it is crucial to incentivize and facilitate salt reduction strategies on a broader scale (5). These strategies can range from policy changes to social marketing campaigns and behavior change interventions. In this context, the advent of digital health presents a wealth of opportunities for cost-effective public health interventions to reduce salt consumption at the population level (6).

The aim of the Research Topic “Digital strategies to reduce salt consumption” is to shed light on the latest research surrounding digital interventions aimed at reducing salt consumption. Specifically, the articles published in this Research Topic collectively provide valuable insights into the use of digital technology to address salt intake reduction and related health concerns across diverse regions.

For digital platforms to be effective and scalable in reducing salt consumption, the following themes warrant special consideration:

1. **Digital engagement**: Smartphone applications and social media tools have emerged as effective channels for educating communities, with a particular focus on school children and families. In Sun et al. and Jarrar et al., researchers explore the potential, and effectiveness, of digital platforms to engage populations in reducing salt intake.
2. **Health behavior associations**: Emphasizing holistic public health approaches that consider the complex interplay of factors such as stress, unhealthy dietary habits, and lifestyle choices is essential for overall wellbeing. Yang et al. and Mahmoud et al. investigate these intricate connections among populations.
3. **Cultural variations and vulnerable populations**: Adapting interventions to suit specific cultural and regional contexts is crucial when designing digital strategies to reduce salt consumption. The study by Al-Qahtani sheds light on unique cultural influences on dietary choices among male university students in Saudi Arabia.
4. **Education and awareness**: Effective mass media campaigns should actively involve the target audience and use tailored communication materials to engage and educate vulnerable subgroups. Capitão et al. focuses on the development of such campaigns to promote healthy eating.
5. **Preventive strategies**: Prioritizing preventive measures in the development and implementation of digital strategies for reducing salt consumption is key. Negesse et al. examine broader health concerns, including dental caries, obesity, and diarrhea prevention, in specific Diarrhea hot-spot regions of Ethiopia, stressing the importance of considering various factors such as residence, educational level, health insurance, and media exposure in designing prevention and control strategies.
6. **Policy recommendations**: The implementation of policies such as food reformulation, warning labels, and communication campaigns can effectively promote healthier eating habits and reduce sodium consumption. Campos-Nonato et al. provide policy recommendations, particularly in the context of Mexico, to reduce table salt intake and monitor salt consumption.

Increasing evidence suggest the potential of digital health interventions, comprising smartphone applications, digital platforms, and legislative interventions, to address the pervasive issue of excessive salt consumption and its associated health risks (7). These digital strategies should be complemented with tailored communication, education, and behavior change initiatives for wider uptake and effectiveness.

In conclusion, digital strategies offer promising avenues to tackle the global burden of high salt consumption (Jarrar et al.). By leveraging technology and implementing evidence-based interventions, we can create scalable and sustainable solutions to reduce population-level salt intake (8). However, further research and collaboration are needed to optimize these strategies and ensure their effectiveness in diverse contexts. By embracing digital health innovations, we can pave the way for a healthier future, reducing the burden of non-communicable diseases and promoting wellbeing for all (9).

## Author contributions

SB conceptualized and wrote the manuscript and approved the submitted version.

## References

[1] WHO. New WHO Benchmarks Help Countries Reduce Salt Intake and Save Lives. (2021). Available from: https://www.who.int/news/item/05-05-2021-new-who-benchmarks-help-countries-reduce-salt-intake-and-save-lives (accessed June 22, 2023). PMC9149714 34078737

[2] Elizabeth L Machado P Zinöcker M Baker P Lawrence M. Ultra-processed foods and health outcomes: a narrative review. Nutrients. (2020) 12:1955. 10.3390/nu12071955 32630022 PMC7399967

[3] Strazzullo P D'Elia L Kandala NB Cappuccio FP. Salt intake, stroke, and cardiovascular disease: meta-analysis of prospective studies. Bmj. (2009) 339:b4567. 10.1136/bmj.b4567 19934192 PMC2782060

[4] Trieu K Neal B Hawkes C Dunford E Campbell N Rodriguez-Fernandez R. Salt reduction initiatives around the world - a systematic review of progress towards the global target. PLoS ONE. (2015) 10:e0130247. 10.1371/journal.pone.0130247 26201031 PMC4511674

[5] Cobb LK Appel LJ Anderson CA. Strategies to reduce dietary sodium intake. Curr Treat Options Cardiovasc Med. (2012) 14:425–34. 10.1007/s11936-012-0182-9 22580974 PMC3612540

[6] Bhaskar S Rastogi A Chattu VK Adisesh A Thomas P Alvarado N. Key strategies for clinical management and improvement of healthcare services for cardiovascular disease and diabetes patients in the coronavirus (COVID-19) settings: recommendations from the REPROGRAM consortium. Front Cardiovasc Med. (2020) 7:112. 10.3389/fcvm.2020.00112 32613010 PMC7308556

[7] Hartin PJ Nugent CD McClean SI Cleland I Tschanz JT Clark CJ. The empowering role of mobile apps in behavior change interventions: the gray matters randomized controlled trial. JMIR Mhealth Uhealth. (2016) 4:e93. 10.2196/mhealth.4878 27485822 PMC4987494

[8] Vargas-Meza J Gonzalez-Rocha A Campos-Nonato I Nilson EAF Basto-Abreu A Barquera S. Effective and scalable interventions to reduce sodium intake: a systematic review and meta-analysis. Curr Nutr Rep. (2023) 12:486–494. 10.1007/s13668-023-00477-w 37226030

[9] Monaco A Palmer K Holm Ravn Faber N Kohler I Silva M Vatland A. Digital health tools for managing noncommunicable diseases during and after the COVID-19 pandemic: perspectives of patients and caregivers. J Med Int Res. (2021) 23:e25652. 10.2196/25652 33464206 PMC7850778

